# Poster Session II - A237 PERFORMANCE OF FECAL CALPROTECTIN AS A SURROGATE MARKER FOR EXTENT OF DISEASE IN CROHN’S DISEASE AND ULCERATIVE COLITIS AT DIAGNOSIS

**DOI:** 10.1093/jcag/gwaf042.237

**Published:** 2026-02-13

**Authors:** J Sgro, C Head, K Jacobson

**Affiliations:** GI, BC Children’s Hospital, Vancouver, BC, Canada; GI, BC Children’s Hospital, Vancouver, BC, Canada; GI, BC Children’s Hospital, Vancouver, BC, Canada

## Abstract

**Background:**

Although endoscopy and biopsies are gold standard, fecal calprotectin is used regularly as both a screening test for IBD as well as routinely after diagnosis to assess for disease activity. When fecal calprotectin is persistently high or elevated from a patient’s baseline, this may be an indication for endoscopy or escalation of medical management. Past research focuses of fecal calprotectin and IBD diagnosis; however, there is limited data looking at the correlation of fecal calprotectin with extent of inflammation and its performance over time.

**Aims:**

To compare fecal calprotectin at diagnosis between Crohn’s disease (CD) and ulcerative colitis (UC) in a pediatric cohort, and to assess the correlation between fecal calprotectin and routine serologic markers with disease extent, defined by Paris classification for CD (L1/L2/L3/L4) and UC (E1–E4).

**Methods:**

Retrospective chart review of all pediatric patients with an endoscopic diagnosis of IBD at BC Children’s Hospital from August 2021 to June 2022. Data collected included clinical notes, endoscopy reports, laboratory results, and imaging. Paris L and E extent were assigned retrospectively. Descriptive statistics and group comparisons were performed; p-values reflect statistical tests comparing relevant subgroups (CD vs UC; L, L/L4 subtypes; E1–E4 subtypes).

**Results:**

Among 100 pediatric IBD patients identified, 31 (31%) had UC and 69 (69%) had CD. Mean fecal calprotectin was similar between UC and CD groups (UC: 3906 ± 3867; CD: 3650 ± 3081; p = 0.76). Within CD, there were no significant differences in hemoglobin, hematocrit, CRP, albumin, or fecal calprotectin across Paris L1, L2, L3, L4 subtypes (p = 0.21, 0.60, 0.12, 0.06, and 0.54, respectively). Within UC, no significant differences were observed across E1–E4 subtypes for the same variables (p = 0.20, 0.14, 0.63, 0.48, 0.48).

**Conclusions:**

In this retrospective study, 31% of patients had UC and 69% Crohn’s, with no significant difference in fecal calprotectin between the groups. Within each diagnosis, fecal calprotectin and common inflammatory markers did not differ by disease extent as defined by Paris classification at diagnosis. These findings contrast with some reports of lower calprotectin in ileal disease and should be interpreted considering the study’s retrospective design, potential selection bias, and limited power for some subgroups. Regular endoscopy remains a key tool for accurate assessment of disease extent in this cohort. Prospective longitudinal work is planned to evaluate fecal calprotectin as a surrogate for disease activity at 1 and 2 years post-diagnosis.

**Funding Agencies:**

None

